# Spot-Weld Service Life Estimate Based on Application of the Interfacial Crack Concept †

**DOI:** 10.3390/ma13132976

**Published:** 2020-07-03

**Authors:** Ružica R. Nikolić, Jelena M. Djoković, Branislav Hadzima, Robert Ulewicz

**Affiliations:** 1 Research Center; University of Žilina, 010 26 Žilina, Slovakia; branislav.hadzima@rc.uniza.sk; 2 Department for Chemistry and Chemical Technology, Technical Faculty in Bor, University of Belgrade, 19210 Bor, Serbia; jdjokovic@tfbor.bg.ac.rs; 3 Department of Production Engineering and Safety, University of Technology, 42201 Czestochowa, Poland; robert.ulewicz@pcz.pl

**Keywords:** spot-welding, thin sheets, interface crack, service life, Paris’s law

## Abstract

In the automotive industry, spot-welding is the most common method of joining components. Thus, determining the service life of spot-welds is of great importance in designing assemblies or structures. It is well-known that lately there has been a trend in the industry toward reducing the fuel consumption and harmful gasses emissions, as well as the weight of structures with the application of the lightweight materials, like aluminum alloys. In this paper, research is presented on the behavior of a spot-weld between the plates made of the two dissimilar materials—aluminum alloy and steel. In addition, the influence of the plates’ thickness and the weld nugget’s diameter on welds’ service life is presented. In this analysis, a concept of the interface crack between the two linear elastic materials was applied. Obtained results show that the plates’ thickness and the nugget’s size, as well as the working load, impose significant influences on the service life of a spot-weld between the two dissimilar materials.

## 1. Introduction

It is the common knowledge that spot-welding is the most frequent method used for joining components in the automotive industry. The car body contains several hundred points of the spot-welds. Of course, there are other methods of executing joints, like with rivets, screws and studs, or gluing, which, to some extent can replace the spot-welding. However, the spot-welding remains the most important method of joining the thin sheets made of construction and low-alloyed steels, since this is a cheap and robust joining method. The strength of a spot-weld in a construction actually determines the integrity of the car construction, carrying performance in exploitation. Though a majority of the spot-welds are loaded by the shear forces only, under certain loading conditions, the peeling forces can appear in some spot-welds, as well as the tensile forces perpendicular to the weld itself. The combination of the stress state and the geometry of the spot-weld’s shape can cause the appearance of the stress concentration, leading to the development of fatigue cracks around the spot-weld. The presence of those fatigue cracks can degrade the carrying performance of the spot-weld and increase the noise and vibrations of the vehicle’s structure. Thus, knowing the fatigue strength of the spot-weld is very important in designing the automotive components.

The spot-welding technique is not used only for joining the thin sheets made of a single material, like the steel sheets, but it can also be applied for joining the thin sheets made of dissimilar materials. Considering the requirements for reducing the fuel consumption, as well as the harmful gasses emission, significant efforts are present in the automotive industry to design and manufacture vehicles made of the lightweight materials. The application of such materials, like, for instance, aluminum alloys, can create much bigger savings of fuels than the application of the traditional materials, such as cast iron or “classical” steel. Nowadays, the idea of joining aluminum and steel in a car body and the vehicle’s components becomes all the more popular. Since, in the car exploitation, a majority of cracks appear on the car body, it is of great importance to estimate the fatigue fractures that are results of the spot-welding, as well as the service life of such a spot-weld.

Many researchers were dealing with various problems concerning the service life of the spot-welds, like determining the stress intensity factor (SIF) for the spot-weld between the plates of different thicknesses or made of different materials, initiation and propagation of a fatigue crack on the spot-weld or in its vicinity, etc. 

Radaj and Shag [1] have determined the stress intensity factor for a spot-welded joint between the two plates of different thicknesses, what served as a basis for estimate of the fatigue strength of the spot-welded samples and components. The same authors were then considering the joining of plates made of dissimilar materials [2]. The specimens were made of aluminum and steel plates and subjected to tension-shear, as well as to the cross-tension. The calculated stress-intensity factors were dependent on elasticity and thickness ratios of the spot-welded plates. 

Sheppard and Strange [3] developed a model of fatigue crack initiation and early crack growth in resistance spot-welds, which is specimen-independent. They formulated a general expression for the structural stress around the nugget, which is dependent only on the loading immediately surrounding the weld. They also investigated the possibility of applying the fatigue crack initiation model for the life estimate of the spot-weld by using the structural response data obtained by the finite element analysis. That was verified for the fatigue crack initiation and early crack growth in cyclically loaded resistance spot-welds. Zhang [4] derived expressions for the stress intensities (notch stress, stress intensity factors and *J*-integral) at spot-welds under typical loading conditions of tensile-shear, cross-tension and coach-peel. Zhang concluded that the behavior of the stress intensities depends on the spot-weld design parameters (nugget diameter and sheet thickness). Those expressions were estimated in terms of the forces and moments transferred by the spot-welds and verified by the finite element analysis.

Though studying a spot-weld fatigue behavior is a complex problem, due to the three-dimensional crack growth, Newman and Dowling [5] were modeling it by a single-degree-of-freedom crack growth problem. That model can enable a design engineer to analyze the fatigue behavior of spot-welded steel sheets without knowledge of metallurgical and geometrical details of a spot-weld. What is needed are only the fatigue properties of the sheet metal; thus, no laboratory tests are necessary to generate the fatigue data, which are specific to spot-welds or weld metal. Newman and Dowling reported that even their simple model produced results that were in good agreement with experimental data. Djoković et al. [6] investigated the behavior of a crack that lay at the interface between two materials (A and B) and was approaching the three materials’ (A, B and C) joint, where exist two interfaces (A/C and B/C). The problem was to define whether the crack would deflect into the first interface (A/C) or into the second interface (B/C). The solution was sought by comparison of energy release rates for the two competing deflections, thus enabling the authors to answer how the crack would behave. The problem of an interface crack approaching the three-material joint could be useful for resolving some problems of micro-cracking during the fatigue loading of construction components. This problem can also arise if the crack between the two zones of the welded joint is approaching the third one, where it is extremely important that the crack would “choose” the less dangerous interface and not propagate in the most critical heat-affected zone.

Pan and Sheppard [7] proposed a fatigue life prediction method based on strains. The FEM (Finite Element Method) modeling results were confirmed by fatigue testing of specimens of three different configurations: tensile shear (TS), modified coach peel (MCP) and modified tensile shear (MTS). It was found that significant yielding occurs in spot-welds even under relatively low loads. Ertas et al. [8] analyzed the fatigue life of spot-welded joints from different aspects. They first analyzed various theoretical solutions for fatigue service life predictions, based on stress and strain approaches, and then they performed experiments on the MTS specimens (manually and automatically welded joints) and, finally, did the FEM modeling by ANSYS program. All the results were compared, and the conclusion on which of the theoretical or numerical predictions was most suitable was drawn based on its best corresponding to experimentally obtained values. Salvini, Vivio and Vullo [9] proposed a procedure for fatigue-life evaluation of the multi-spot-welded structures, using the equivalent radial stress (ERS). The ERS is based on a closed-form solution of a theoretical bi-dimensional model of the spot-weld area, under various types of loading conditions. They concluded that, through the use of the ERS-N curves (N is a number of load cycles), fatigue data collected on different joint geometries can be successfully combined. One of the main aspects evidenced is that progressive damage deeply influences the fatigue behavior of a welded structure. They also pointed that, by modeling the experimental data, “it is possible to choose the end life criterion as desired, no matter what is the experimentalist’s selection”.

Fujii et al. [10] have carried out the fatigue tests and the FEM analysis of the spot-welded and spot-weld-bonded joints of mild steel and ultra-high-strength steel plates. The fatigue tests were carried out on both types of specimens, under the sinusoidal cyclic load, either to fracture or to 10^7^ load cycles (if the specimen was not broken). They concluded that the fatigue strength of the spot-weld-bonded joints is higher than that of the spot-welded joints and that the interfacial debonding propagates from the adhesive edge to a nugget edge, after which the fatigue crack initiates at the nugget edge. The stress intensity factors at a nugget edge are low at the beginning of fatigue and increase with the progress of interfacial debonding. The fatigue strength of the spot-weld-bonded specimens is improved because the stress concentration at the nugget edge was reduced by adhesive bonding during the large part of fatigue life. Miletić et al. [11] and Ilić et al. [12] analyzed certain properties of the welded joints of the high-strength low-alloyed steels (HSLA). The comparison was performed of the two different welding procedures, to investigate their influence on impact toughness of the welded joints. The two compared procedures consisted of the MIG (Metal Inert Gas) or MMA (Manual Metal Arc) process used for the root passes, with the MAG (Metal Active Gas) process used for the filling and covering passes. Investigation included determination of the joints’ fracture energy when the notch was placed in different zones of the welded joint. The general conclusion was that applying the MMA/MAG welding provides joints with more favorable characteristics. 

Shariati and Nejad [13] investigated, experimentally and numerically, the fatigue behavior of the metallic welded U-shape specimens subjected to cyclic loading. The tests were performed on the two sets of the spot-welded specimens of different sizes, and the fatigue tests were conducted under various cyclic loads. The crack propagation was numerically examined by use of the stress intensity factors obtained by the FEM analysis. They used the modified Paris and Forman–Newman–De Koning models to estimate the fatigue-crack growth rate. Their results indicate that the fatigue life of specimens decreases with any increase of the load level. Nový et al. [14] investigated the fatigue resistance of nine structural steels, including high-strength steels, DOMEX 700MC, HARDOX 400, HARDOX 450 and 100Cr6, at high-frequency cyclic loading, in the region of loading cycles number that ranged from 2 × 10^6^ to 2 × 10^9^. Their experimental results led to conclusions that, in tested steels, a continuous decrease of fatigue strength was observed with an increase of number of loading cycles, with the average value of ratio of strengths at 10^9^ and 10^6^ cycles of 0.69. They also concluded that values of conventional fatigue limit (usually referred to 2 × 10^6^ ÷ 10^7^ cycles) are overestimated and, therefore, do not meet demands of safety of structural parts. Thus, those facts must be taken into account during the design phase of structural parts in the ultra-high region of loading cycles, what also must include design of the spot-welded joints. Yuan et al. [15] extensively investigated all the properties of the spot-welded joint of the two dissimilar steels, ultra-low carbon DP600 steel and galvanized DC54D steel. They studied the development of the weld nugget, whose diameter increased with an increase of the heat input and the development of the microstructure and macrostructure of the heat-affected zone, fusion zone and the weld meta; they measured micro hardness in different zones of the welded joint and studied types of fractures of the joined steel plates, as well.

The spot-welding between the two plates made of dissimilar materials is considered, in this paper, as a problem of an interface crack between the two elastic layers with application of the linear elastic fracture mechanics assumptions (the material is isotropic and linear elastic; the small-scale yielding is present; the stress field near the crack tip is calculated by using the theory of elasticity; the crack would propagate if the stresses near the crack tip exceed the material fracture toughness).

## 2. Interface Crack Approach for the Problem Formulation

**Figure 1:**
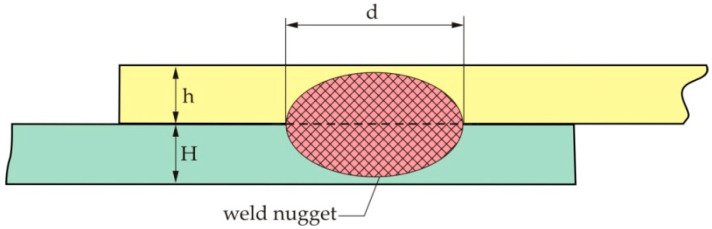
The geometry of the overlap spot-welded joint.

Figure 1 shows the geometry of the overlap spot-weld of the two plates of thicknesses *h* and *H*, respectively, made of the two different materials. The nugget diameter is *d*. An assumption is that the fatigue crack is propagating along the interface.

The fatigue fracture appears due to the long-term exposure of a component to a cyclic loading. There are three phases of the fatigue fracture: crack initiation, crack propagation (growth) and fracture. The fatigue service life of a structural element can be predicted through the application of the fracture mechanics concept, based on the fact that the stress field around the crack tip can be described by a single parameter, by the stress intensity factor *K* or, in the case of the cyclic loading, by change of the stress intensity factor, *ΔK* (the difference between the stress intensity factor values at the maximal and minimal load).

The unstable crack growth appears when the stress intensity factor, $K_{I}$, becomes bigger than the experimentally determined material characteristics, the fracture toughness, $K_{Ic}$, i.e., for $K_{I}\;>\;K_{\;Ic}\;.$ The crack growth equation gives the relationship between the crack length increase, *Δa*, and increase of the loading cycles’ number, *ΔN*. Paris and Erdogan established, in 1963, that variation of the stress intensity factor can describe the sub-critical crack growth in the fatigue loading conditions in the same way as the stress intensity factor describes the critical or the fast fracture [16]. They determined that the crack growth rate is a linear function of the stress intensity factor variation in the logarithmic diagram:

(1) $$\frac{da}{dN}=C{\left(\Delta K\right)}^{m},$$

where *a* is the crack length that varies from the initial value, $a_{i}$, to the critical value, $a_{cr}$, which leads to fracture; *N* is the number of loading cycles; *C* and *m* are the material constants; and $\Delta K=K_{max}-K_{min}$ is the stress intensity range, i.e., the difference between the stress intensity factors at maximum and minimum load. 

Dependence *da/dN* is in the log–log coordinate frame represented by the S-shaped curve (sigmoidal curve). The curve asymptotically approaches the limits of the crack growth *ΔK_th_* and *ΔK_C_*. The threshold of the stress intensity factor, *ΔK_th_*, is the limit value, below which the crack does not propagate. The fracture appears at values *ΔK_C_*.

The remaining service life is obtained by integration of Equation (1) as follows:

(2) $$N=\int_{a_{i}}^{a_{cr}}\frac{da}{C{\left(\Delta K\right)}^{m}}.$$

**Figure 2:**
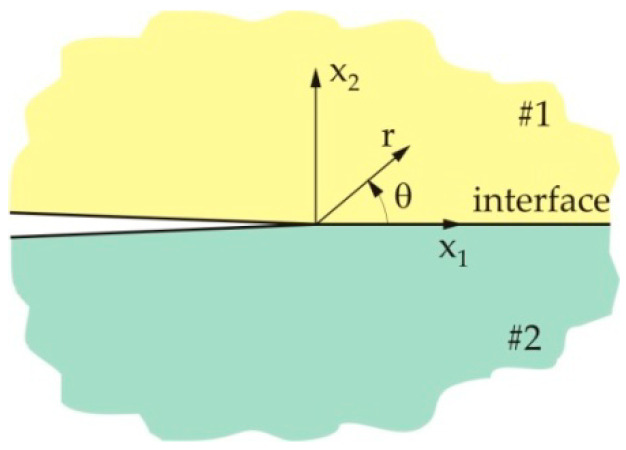
Geometry of a crack at the interface between the two dissimilar materials.

The problem presented in Figure 1 is here considered as a problem of a crack at the interface between the two dissimilar layers. To obtain the stress and strain fields at the tip of the crack at the interface between the two dissimilar materials, one considers the two-dimensional elastic problem, which is presented in Figure 2. Materials 1 and 2 are joined along the positive part of the $x_{1}$ axis, while the crack lies along the negative part of the $x_{1}$ axis. The $x_{2}$ axis is perpendicular to the crack plane and the interface. The polar coordinates are denoted as *r* and *θ*. 

Forces at the interface at a distance *r* ahead of the crack tip are characterized by the complex stress intensity factor,$K=K_{1}+iK_{2}$ and have the form defined by Rice, Suo and Wang [17]:

(3) $${\left(\sigma_{22}+i\sigma_{12}\right)}_{\theta =0}=\frac{(K_{1}+iK_{2})r^{i\varepsilon }}{\sqrt{2\pi \;r}}.,$$

Parameter *ε* is called the bi-elastic constant or the oscillatory index. It is a characteristic of the interface and is determined as follows [18]:

(4) $$\varepsilon =\frac{1}{2\pi }\ln\left(\frac{1-\beta }{1+\beta }\right),$$

where *α* is one of the two Dundurs’ parameters, defined as follows [19]:

(5) $$\alpha =\frac{\mu_{2}(\kappa_{1}+1)-\mu_{1}(\kappa_{2}+1)}{\mu_{2}(\kappa_{1}+1)+\mu_{1}(\kappa_{2}+1)},\;\beta =\frac{\mu_{2}(\kappa_{1}-1)-\mu_{1}(\kappa_{2}-1)}{\mu_{2}(\kappa_{1}+1)+\mu_{1}(\kappa_{2}+1)},$$

where $\mu_{i}$ is the shear modulus, and $\kappa_{i}=3-4\nu_{i}$ holds for the plane strain state, while $\kappa_{i}=(3-\nu_{i})/(1+\nu_{i})$ holds for the plane stress state; $\nu_{i}$ is the Poisson’s ratio. Subscripts *i* = 1, 2 refer to properties of materials above and below the interface, respectively.

Unlike the case of the homogeneous material, where there are two separate stress intensity factors for Mode I and Mode II of the crack loading, for the case of an interface crack, there is a complex stress intensity factor, $K=K_{1}+iK_{2}$, for the planar modes, which are coupled. The interface stress intensity factors are defined in such a way that $K_{1}\to K_{I}$ and $K_{2}\to K_{II}$ for the case when the materials across the interface are identical. When *β* = 0 and *ε* = 0,$K_{1}$ then represents a measure of the normal component of the stress singularity, which acts on the interface, while $K_{2}$ measures the shear component, with the usual definition of the stress intensity factors.

**Figure 3:**
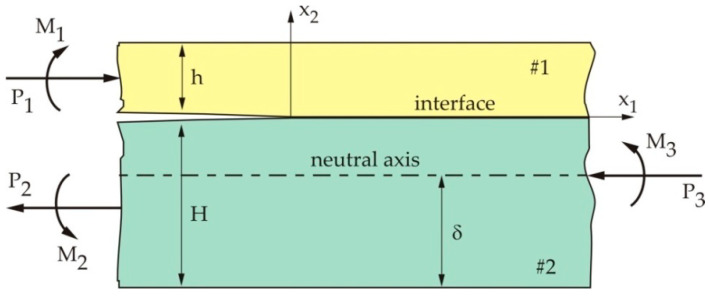
Semi-infinite crack at the interface between the two layers.

Figure 3 shows the semi-infinite crack at the interface between the two dissimilar homogeneous isotropic elastic layers under the general loading conditions (*P* denotes the acting forces, while *M* denotes the moments).

Based on the analysis by Hutchinson and Suo [20], far ahead of the crack tip, this two-layered sample can be considered as a composite beam. The neutral axis lies at a distance of δ=Δh from the bottom of Layer 2, where one gets the following:

(6) $$\Delta =\frac{1+2\Sigma \eta +\Sigma \eta^{2}}{2\eta (1+\Sigma \eta )},\;$$

with η=h/H being the relative thickness of the layers and $\Sigma ={\bar{E}}_{1}/{\bar{E}}_{2}$ being the ratio of reduced elasticity moduli, where ${\bar{E}}_{1}=E_{1}/(1-\nu_{1}^{2})$ and ${\bar{E}}_{2}=E_{2}/(1-\nu_{2}^{2})$ are valid for the plane strain conditions. Variables $E_{1}$ and $E_{2}$ represent the Young’s elasticity moduli of Layers 1 and 2, respectively.

The two-layered sample is in conditions of pure tension combined with pure bending. The only stress component that is not equal to zero is $\sigma_{11}$. The corresponding strain component is a linear function of the distance from the neutral axis:

(7) $$\varepsilon_{11}=-\frac{1}{{\bar{E}}_{2}}\left(\frac{P_{3}}{hA}+\frac{M_{3}}{h^{3}I}x_{2}\right),\;$$

where the dimensionless values of the cross-section area and the inertia moment are defined as follows:

(8) $$A=\frac{1}{\eta }+\Sigma ,\;I=\Sigma \left[{\left(\Delta -\frac{1}{\eta }\right)}^{2}-\left(\Delta -\frac{1}{\eta }\right)+\frac{1}{3}\right]+\frac{\Delta }{\eta }\left(\Delta -\frac{1}{\eta }\right)+\frac{1}{3\eta^{3}}.$$

Based on the linearity of the problem and dimensional analysis, the complex stress intensity factor can be determined as follows:

(9) $$K=K_{1}+iK_{2}=h^{-i\varepsilon }\sqrt{\frac{1-\alpha }{1-\beta^{2}}}\cdot \left(\frac{P}{\sqrt{2Uh}}-ie^{i\gamma }\frac{M}{\sqrt{2Vh^{3}}}\right)e^{i\omega },\;$$

where *P* and *M* are the linear combinations of the applied loads, determined by the following:

(10) $$P=P_{1}-C_{1}P_{3}-\frac{C_{2}M_{3}}{h},\;M=M_{1}-C_{3}M_{3},\;$$

Meanwhile, the geometric factors are determined as follows:

(11) $$C_{1}=\frac{\Sigma }{A},\;C_{2}=\frac{\Sigma }{I}\left(\frac{1}{\eta }+\frac{1}{2}-\Delta \right),\;C_{3}=\frac{\Sigma }{12I},\;$$

and

(12) $$U=\frac{1}{1+\Sigma (4\eta +6\eta^{2}+3\eta^{3})},\;V=\frac{1}{12(1+\Sigma \eta^{3})},\;\gamma =\arcsin(6\Sigma \eta^{2}(1+\eta )\sqrt{UV}),$$

The angle ω is a function of Dundurs’s parameters and the relative layer’s thickness, i.e., ω≡ω (α,β,η). This function is defined by Veljkovic and Nikolic [21], based on the solution of the elasticity problem and the tabular results of Suo and Hutchinson [22], and it reads as follows:

(13) $$\omega =\frac{1-\eta }{1+\eta }\cdot \sqrt{\frac{\beta (1-\alpha )}{\alpha -\beta^{2}}}.\;$$

If *h* is the reference length variable, for the real and imaginary part of the complex stress intensity factor, one can write the following:

(14) K1=Re(Khiε)=1−α1−β2⋅[P2Uhcosω+M2Vh3sin(ω+γ)],K2=Im(Khiε)=1−α1−β2⋅[P2Uhsinω−M2Vh3cos(ω+γ)].

The load phase angle, as a measure of the relative value of the Mode 2 with respect to Mode 1, for the reference length, *h*, ahead of the crack tip, according to Reference [20], can be written as:

(15) $$\psi =arctg\frac{K_{2}}{K_{1}}=arctg\left[\frac{\xi \sin\omega -\cos(\omega +\gamma )}{\cos\omega +\sin(\omega +\gamma )}\right],\;$$

where $\xi =\frac{Ph}{M}\sqrt{\frac{U}{V}}$.

Energy release rate, within the concept of the plane strain state, can be calculated as a difference in energies in material far ahead and far behind the crack tip [20]:

(16) $$G=\frac{1}{2{\bar{E}}_{1}}\cdot \left[\frac{P^{2}}{Uh}+\frac{M^{2}}{Vh^{3}}+2\frac{PM}{h^{2}\sqrt{UV}}\cdot \sin\gamma \right].$$

## 3. Results and discussion

**Figure 4:**
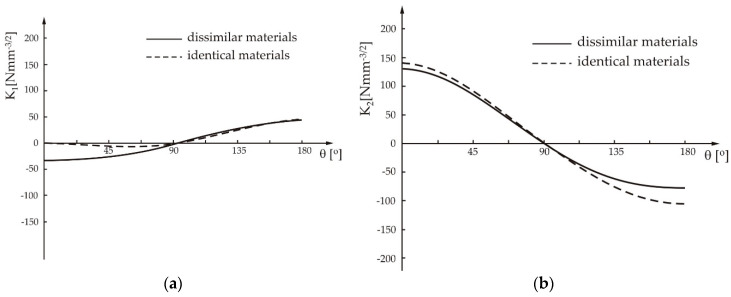
Stress intensity factors variation with angle *q* around the weld nugget for the spot weld of identical and dissimilar materials: (**a**) *K_1_* and (**b**) *K_2_*.

Based on Equations (1), (2) and (14), using the symbolic programming routine Mathematica^®^, an estimate of the fatigue service life of a spot-weld was performed. The following data were taken into account in calculations: $E_{1}=0.75\times 10^{5}{N/\mathrm{mm}}^{2},$$E_{2}=2.1\times 10^{5}{N/\mathrm{mm}}^{2},$$\nu_{1}=\nu_{2}=0.3,$ *h* = 0.5 mm and *H* = 2.0 mm. The spot-weld diameter was taken to be 3, 5 and 8 mm. The material constants for calculation of the remaining fatigue service life, according to Paris’s law, were *m* = 2.75 and $C=1.95\times 10^{-12}.$ Figure 4 shows the variations of the stress intensity factors, $K_{1}$ and $K_{2}$, around the weld’s nugget with diameter of 5 mm, for the case that the sheets are made of the same or dissimilar materials.

As can be seen from Figure 4, when the two dissimilar materials are spot-welded, stress intensity factor, has negative values, which can positively affect the crack closure. In addition, it can be seen that the absolute value of the stress intensity factor, $K_{1}$ is smaller than the value of $K_{2}$.

**Figure 5:**
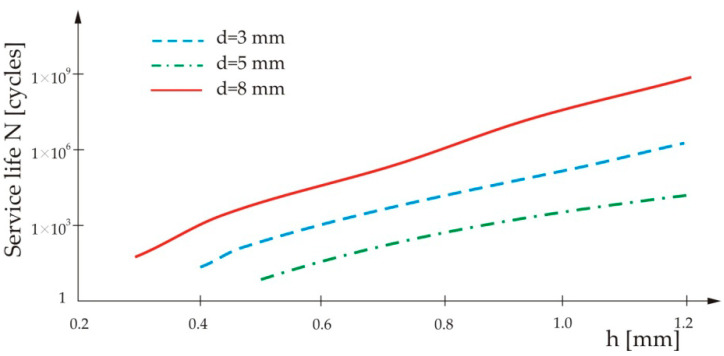
Influence of Thin Sheet 1’s thickness on the service life of the spot-weld for three different nugget diameters.

Figure 5 shows the influence of Thin Sheet 1’s thickness and the nugget’s diameter on the service life of the spot-weld.

From Figure 5, one can see that the service life of the spot-weld increases with an increase of Thin Sheet 1’s thickness, as well as with increase of the spot-weld diameter.

**Figure 6:**
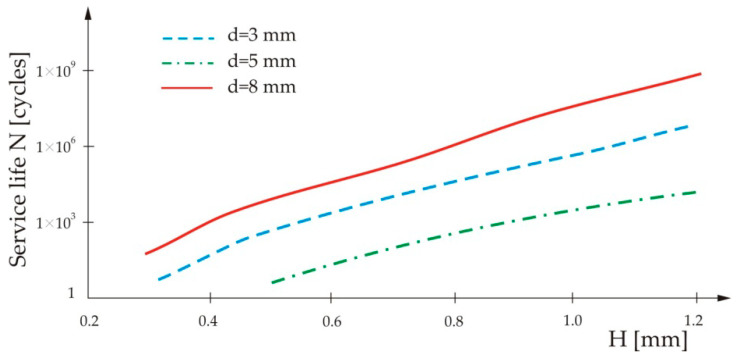
Influence of Thin Sheet 2’s thickness on the service life of the spot-weld for three different nugget diameters.

Figure 6 shows the influence of Thin Sheet 2’s thickness, *H*, and the nugget diameter, *d*, on the service life of the spot-weld.

From Figure 6, it can be seen that the service life of the spot-weld increases with increase of Thin Sheet 2’s thickness, as well as with increase of the nugget diameter.

**Figure 7:**
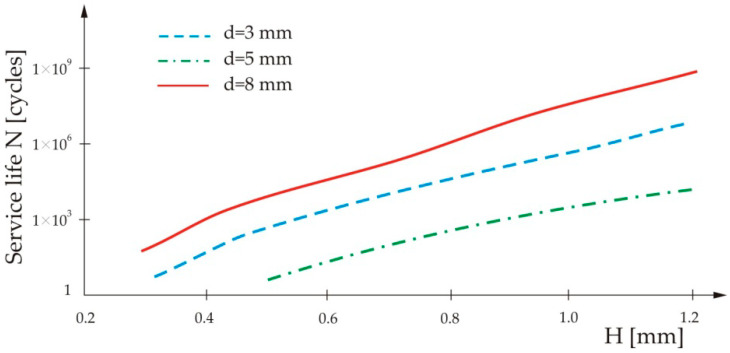
Influence of loading on the service life of the spot-weld for three different nugget diameters.

Figure 7 presents the result of the analysis of the loading influence on the service life of the spot-weld for three different nugget diameters.

From Figure 7, it can be seen that the service life of the spot-weld decreases with increase of the load.

**Figure 8:**
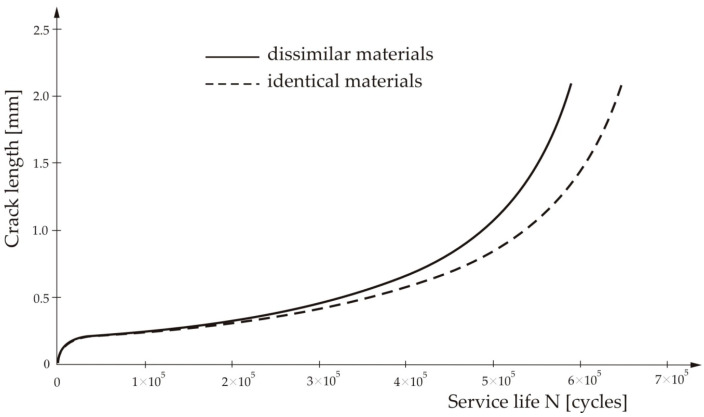
Number of load cycles needed for the crack propagation for the two cases of welded joints.

Figure 8 shows the number of loading cycles needed for the crack propagation for the case that the welded plates are made of the same or dissimilar materials.

From Figure 8 can be seen that the crack would not propagate (or rather would propagate very little) until the number of cycles reached 30,000. Then, its length increased almost linearly, up to 40,000 cycles, when the crack growth rate started to increase faster and the crack became unstable. It can also be seen that the service life of the spot-welded joint of the two identical materials is bigger than that of the joint of the two dissimilar materials. That is expected due to existence of differences in the mechanical properties of the two materials, primarily resulting from the difference in strength and the thermal expansion coefficients. 

## 4. Conclusions

Considering that, in the vehicle’s exploitation, a majority of cracks appear on the car body, it is of great importance to analyze the fatigue fractures that appear in the spot-welded joints or are the consequence of those joints. The analysis must include an estimate of the remaining service life of such joints, subsequently to the welded structure as a whole.

In this paper is considered the spot-welded joint between the two plates made of the two dissimilar materials, aluminum alloy and steel. Joints of those two materials appear most frequently when assembling the parts of vehicles. Three different diameters of the weld nugget were considered, i.e., 3, 5 and 8 mm.

An analysis was performed based on application of the linear elastic fracture mechanics concept to an interface crack between the two elastic layers. The fatigue service life of the spot-welded joint was estimated based on the Paris law for cyclically loaded structures.

Based on obtained results, it is concluded that the diameter of the weld nugget and the thickness of the two layers constituting the interface, impose significant influence on the service life of the spot-welded joint. The service life increases with an increase of thickness of either layer, as well as with an increase of the nugget diameter; meanwhile, it decreases with an increase of the applied load to the spot-welded joint.

The crack propagation behavior was analyzed, and it was concluded that the crack practically does not extend until the number of loading cycles reaches 30,000, when the crack length starts to increase linearly up to about 40,000 cycles, after which the crack growth becomes unstable. 

The crack propagation rate was also analyzed, and a comparison was performed for the case when the spot-welded joint was executed between the two considered dissimilar materials and for the case when the joint is executed between the two identical materials. The service life is bigger for the joint of the two identical materials. That is understandable, since in the case of the dissimilar materials, additional residual stresses appear due to difference in their mechanical properties, primarily their strength, as well as due to the difference in their thermal expansion coefficients. This latter influence was not considered in this paper, but it will be a subject of the further research.
